# Does Farming Have an Effect on Health Status? A Comparison Study in West Greece 

**DOI:** 10.3390/ijerph10030776

**Published:** 2013-02-26

**Authors:** Konstantinos Demos, Eleni Sazakli, Eleni Jelastopulu, Nikolaos Charokopos, John Ellul, Michalis Leotsinidis

**Affiliations:** 1 Rural Medical Dispensary of Dokimio, Health Centre of Thermo, Thermo, GR-30008, Greece; E-Mail: kntemos@upatras.gr; 2 Laboratory of Public Health, Medical School, University of Patras, Rio, Patras, GR-26504, Greece;E-Mails: elsazak@upatras.gr (E.S.); jelasto@upatras.gr (E.J.); 3 Department of Pulmonology, General Hospital of Pirgos, Pirgos, GR-27100, Greece; E-Mail: charok67@hotmail.com; 4 Department of Neurology, University Hospital of Patras, Rio, Patras, GR-26504, Greece; E-Mail: ellul@upatras.gr

**Keywords:** farming, health impairments, haematological and biochemical alterations, neurobehavioral tests, hypertension

## Abstract

Investigating the health status of agricultural workers is a challenging goal. Contradictory outcomes concerning farmers’ health are reported in the literature. In this cross-sectional study, certain clinical and neurobehavioral health outcomes were compared between farmers and non-farmers living in the same rural area. Farmers (328) and non-farmers (347), matched *per* age and sex, were selected randomly in an agricultural area in West Greece. Both groups underwent haematological and biochemical examinations and were administered two neurobehavioral tests, namely the Mini-Mental State Examination (MMSE) and the Montgomery-Åsberg Depression Rating Scale (MADRS). Sociodemographic, personal medical, nutritional and lifestyle data were recorded. According to personal statements, farmers suffered from hypertension, cardiovascular, orthopaedic and ENT problems in higher frequency. Haematocrit, haemoglobin and serum cholinesterase’s activity were found to be lower among farmers. Lower prevalence of hypertension and better performances on MMSE and MADRS tests were recorded in young farmers in relation to young non-farmers, while these findings were reversed in older ages. Odds Ratios were calculated through multivariate logistic regression models. Factors affecting these impairments remain to be clarified.

## 1. Introduction

Farmers and agricultural workers are believed to be healthier and have lower morbidity and mortality rates than non-farming rural and urban populations [1,2,3,4,5]. This fact has been reported as possibly attributable to a healthier lifestyle, especially with respect to drinking and smoking habits, more intensive physical activity and a healthier diet followed by farmers compared to non-farming populations [1,6,7]. 

On the other hand, farming itself and farming-related tasks entail significant hazards to the health and well-being of farmers. Although not well appreciated, farming is among the most hazardous of occupations [8]. Farmers work long hours in hazardous and physically demanding work environments [9]. Health impairments observed on farmers is a highly controversial issue and many studies have focused on agricultural work-related factors that may have a health impact [1,8,10,11,12,13]. Agricultural workers are exposed to a wide range of occupational hazards, such as ergonomic stress, sunlight, viruses, inorganic dust, pesticides and other chemicals [10]. All these exposures have been investigated as possible risk factors for the reported adverse health effects in farmers including musculoskeletal disorders, respiratory diseases, injuries, cardiovascular diseases, pesticides poisoning and neurological dysfunction [9,10,14]. 

Moreover, stress in farmworkers has been recently recognized as an important public health concern. Stressors inherent in farm work and lifestyle, such as uncertain and fluctuating economic prospects are associated with poor physical and mental health outcomes and result in deleterious effects on cognitive function, depression and high rates of suicide [10,13,15]. 

In Greece, about 20% of the labour force population is engaged in agriculture. While agriculture is one of the most promising sectors of the Greek economy, little interest has been demonstrated in studying health and safety of the farming population. Farmers in Greece are involved in all kind of agricultural tasks [16].

The aim of the present study was to assess the health status, as this is reflected by the haematological and biochemical parameters and neurobehavioral and cognitive function, among farming population in relation to non-farmers living under similar environmental conditions. 

## 2. Materials and Methods

A two-year (2008–2009) cross sectional study was conducted in the prefecture of Aitoloakarnania, an agricultural area in West Greece, where about 30% of the total population deals with various cultivations. It is worth noting that the majority of the farming population in this area is indigenous and non-immigrants. The study population was acquired from 36 rural communities of the prefecture. The number of farmers selected in each community was proportional to its total agricultural population. Farmers were selected randomly from the community’s farmer registry. To participate in the study the farmers had to be at least thirty-five years old, so as to satisfy the criterion of long-term farming (at least 15–20 years of farming), given that farmers start dealing with intensive agricultural work at the age of 15 to 20 years old. Non-farmers had to live in the same communities, not to be occupationally involved in farming and to match the recruited farmers *per* sex and age category. Non-farmers were selected randomly from the local telephone directories. A first phone contact took place, in order to explain the aims of the study to the recruits. The response rate was 85%. Finally 328 farmers (196 males, 132 females) and 347 non-farmers (208 males, 139 females) were recruited.

Furthermore, lifestyle characteristics *i.e*., physical activity (type and duration of exercise), smoking habits (packs per day), alcohol consumption (frequency and kind of drinks per day) and coffee consumption (cups per day) were recorded. The smokers were classified as moderate (up to one pack or 20 cigarettes per day) and as heavy (more than one pack per day). Based on daily intake of ethyl alcohol the subjects were classified as abstainer, light, moderate and heavy drinkers [17]. Weekly consumption frequencies of major food groups *i.e*., meat, fish, vegetables, legumes, dairy products, potatoes, candies and fruits were assessed by a 7-day food frequency questionnaire. 

Following the home visit, all subjects were invited to the local health centre or rural dispensary to conduct clinical and laboratory examinations. Measurements of body weight and height, systolic (SBP) and diastolic blood pressure (DBP) and blood examination to determine various biochemical parameters were performed. The participants were classified as hypertensive if SBP >140 or DBP >90.

Finally, the participants were administered two validated questionnaires, the Mini-Mental State Examination (MMSE) and the Montgomery-Åsberg Depression Rating Scale (MADRS). The MMSE is a measure of cognitive components, namely orientation, linguistic and visual functions, with possible scores ranging from 0 to 30 [18]. Lower performances indicate cognitive deficit. The cutoff value was set at ≤24. This cutoff has been evaluated previously in detecting cognitive impairment by General Practitioners with sufficient accuracy [19].

The MADRS is not a diagnostic tool, but can be used to evaluate mood, anxiety, sexual function, appetite, sleep, functional status, ability to think and concentrate, existing physical symptoms or physical complaints, as well as general psychiatric and potential distress [20]. The overall score on the MADRS can range between 0 and 60, with higher scores representing more severe depression symptoms. Cut-off point was set at ≤6 to define subjects with no symptoms of depression. Higher MADRS scores were characterized as “any grade of depression” [21].

### 2.1. Ethics Approval

The study was approved by the plenary meeting of the Medical School of Patras and the Ethics Committee of the University Hospital of Patras.

### 2.2. Statistical Analysis

Statistical analysis was performed by SPSS v.17 statistical software (SPSS Inc., Chicago, IL, USA). Continuous variables are presented by range and median and categorical variables as frequencies. Mann and Whitney U test was employed to compare groups. Differences in proportions were tested using either the Pearson chi square test or Fisher’s exact test depending on cell frequencies. The statistical significance level was set at a = 0.05. 

In order to investigate the effects of the various characteristics of the study population as well as the nutritional and other habits on MMSE, MADRS and hypertension, multivariate logistic regression models were applied. The independent variables were dichotomized at the respective cut-off values. The coefficients of retained parameters and/or interactions of logit functions were calculated. Coefficients with a p < 0.100 were kept in the model. Because of the hierarchy principal, parameters in statistical significant interaction terms were retained in the model independently of their significance. 

## 3. Results

### 3.1. Study Population

The characteristics of the two examined groups, farmers and non-farmers, are shown in Table 1. 

**Table 1:** Characteristics of the study population.

		Farmers			Non-farmers		
		Total	Males	Females	Total	Males	Females
		*n = 328*	*n= 196*	*n = 132*	*n = 347*	*n = 208*	*n = 139*
Age distribution (%)	<40	18.6	18.4	18.9	18.4	18.3	18.7
	40–49	25.6	25.5	25.8	25.6	25.5	25.9
	50–59	27.4	27.6	27.3	27.1	26.9	27.3
	60–69	18.3	18.4	18.2	19.3	19.7	18.7
	70+	10.1	10.2	9.8	9.5	9.6	9.4
Family Status (%)	Married	86.6	81.6	93.9	88.1	81.6	97.7
	Single	10.1	14.3	3.8	11.6	17.9	2.3
	Other	3.4	4.1	2.3	0.3	0.5	-
Number of children (%)	0	11.0	15.8	3.8	13.6	20.7	3.1
	1	6.1	8.7	2.3	11.8	18.0	3.1
	2	27.8	28.6	26.5	29.4	25.3	34.6
	3	31.4	27.0	37.9	27.1	19.3	38.5
	>3	23.8	19.9	29.5	18.2	16.6	20.8
Educational Level (%)	Illiterate ^1^	10.1	10.7	9.1	7.8	6.0	10.8
	Elementary	83.2	82.1	84.8	60.5	51.6	75.4
	Secondary	5.5	5.1	6.1	15.6	19.4	9.2
	Higher	1.2	2.0	-	16.1	23.0	4.6
Income Level (%)	<9,000 €	42.1	20.4	74.3	43.0	43.8	41.6
	9,000–15,000 €	31.4	38.8	20.5	35.1	32.7	39.2
	>15,000 €	26.5	40.8	5.3	21.9	23.5	19.2
Body Mass Index (%)	Normal	17.4	16.8	18.2	16.7	12.9	23.1
	Overweight	56.7	52.6	62.9	56.5	56.7	56.2
	Obese	25.9	30.6	18.9	26.8	30.4	20.8
Smoking (%)	Non smoker	59.45	36.73	93.18	55.04	39.17	81.54
	Medium	14.33	19.90	6.06	22.48	26.27	16.15
	Heavy	26.22	43.37	0.76	22.48	34.56	2.31
Alcohol Consumption (%)	Abstainer	55.18	31.12	90.91	56.77	36.87	90.00
	Moderate	8.54	11.73	3.79	11.53	14.75	6.15
	Heavier	36.28	57.14	5.30	31.70	48.39	3.85
Coffee Consumption (%)	No coffee	4.57	6.12	2.27	5.19	6.45	3.08
	1 cup/day	14.33	6.63	25.76	7.49	7.37	7.69
	2 cup/day	62.80	64.80	59.85	66.28	61.29	74.62
	≥3 cup/day	18.29	22.45	12.12	21.04	24.88	14.62

^1^ up to two years of elementary school.

Differences in the educational level were observed, with farmers showing extremely low percentages, especially regarding secondary or higher degrees. Farmers were more likely to have three or more children in comparison to the non-farmers. Furthermore, higher income levels were observed in male farmers than in male non-farmers, whereas the opposite was observed for females. The prevalence of obesity was very high for both farmers and non-farmers. The percentage of heavy smokers was higher in male farmers while the percentage of female smokers was higher among non-farmers for both categories. Similarly, consumers of two or more cups of coffee per day were higher among female non-farmers. The consumption frequencies of major food groups *i.e*., meat, fish, vegetables, legumes, dairy products, potatoes, candies and fruits did not differ between farmers and non-farmers (data not shown). 

### 3.2. Comorbidities

Comorbidities, according to participants’ statement, are presented in Table 2. Health conditions occurring more frequently among farmers in comparison with non-farmers were hypertension and other cardiovascular disorders as well as ear-nose-throat (ENT) and orthopaedic problems. Moreover reproduction miscarriages were more frequent among farmers than non-farmers (9/123, 3/127 respectively) although at a non-significant level (p = 0.137, Fisher’s exact test).

**Table 2:** Morbidities according to subjects’ statement.

	Farmers *(n = 328)*		Non-farmers *(n=347)*		
	n	*(%)*	n	*(%)*	*p*
None	23	*(7.0)*	40	*(11.5)*	***0.044***
Gastrointestinal diseases ^1^	39	*(11.9)*	44	*(12.7)*	*0.755*
Rheumatoid arthritis	3	*(0.9)*	3	*(0.9)*	*1.000*
Polymyalgia rheumatica ^2^	7	*(2.1)*	9	*(2.6)*	*0.695*
Osteoporosis	27	*(8.2)*	24	*(6.9)*	*0.518*
Thyroid gland disorders ^3^	15	*(4.6)*	23	*(6.6)*	*0.247*
Diabetes mellitus	29	*(8.8)*	32	*(9.2)*	*0.863*
Hypertension	89	*(27.1)*	44	*(12.7)*	***<0.001***
Other cardiovascular disorders ^4^	29	*(8.8)*	16	*(4.6)*	***0.028***
Dislipidemia ^5^	45	*(13.7)*	47	*(13.5)*	*0.947*
Respiratory Diseases ^6^	27	*(8.2)*	27	*(7.8)*	*0.829*
ENT diseases ^7^	19	*(5.8)*	8	*(2.3)*	***0.021***
Dermatological diseases ^8^	14	*(4.3)*	20	*(5.8)*	*0.375*
Ophthalmological diseases ^9^	21	*(6.4)*	17	*(4.9)*	*0.397*
Various orthopaedic disorders ^10^	95	*(29.0)*	43	*(12.4)*	***<0.001***
Brucellosis	3	*(0.9)*	1	*(0.3)*	*0.360*
Renal diseases ^11^	11	*(3.4)*	12	*(3.5)*	*0.940*
Parkinson’s disease	3	*(0.9)*	2	*(0.6)*	*0.678*
Psychosis	3	*(0.9)*	4	*(1.2)*	*1.000*
Depression	24	*(7.3)*	33	*(9.5)*	*0.306*
Cancer ^12^	7	*(2.1)*	5	*(1.4)*	*0.496*

^1^ indigestion, gastric ulcers, intestine problems (infections, polyps, inflammatory bowel diseases). ^2^ clinical syndrome characterized by proximal myalgia and stiffness of the hip and shoulder that last more than one hour after awaking in the morning. ^3^ hyper- and hypothyroidism. ^4^ coronary disease, congestive heart failure, myocarditis, atrial fibrillation and other cardiac arrhythmias, strokes, peripheral vascular diseases. ^5^ including a history of dislipidemia and/or the taking of dislipidemic drugs. ^6^ chronic obstructive pulmonary disease (emphysema, chronic bronchitis), pulmonary infections, bronchial asthma. ^7^ mainly hearing loss, recurrent otic infections, chronic rhinitis, chronic sore throat. ^8^ mainly chronic dermatitis, eczema. ^9^ recurrent ocular infections, conjunctivitis, glaucoma. ^10^ bone fractions, sprains, other joins’ injuries. ^11^ renal failure, pyelonephritis, nephrolithiasis. ^12^ cancer of all types and focus.

### 3.3. Haematological and Biochemical Blood Examinations

The haematological blood examinations for farmers and non-farmers are presented by sex, as some basic haematological parameters like haematocrit and haemoglobin differ considerably between men and women (Table 3). Haematocrit and haemoglobin appeared to be lower among both males and females farmers while the number of WBC was increased in female farmers. Similarly, the MCH index was higher among female farmers.

The biochemical blood examinations are presented in Table 4. Potassium, calcium, SGOT, LDH, bilirubin, total proteins and CRP’s levels were higher among farmers. Triglycerides’ levels were also higher among farmers, while serum cholinesterase’s activity seems to be depressed among farmers.

**Table 3:** Haematological blood examinations.

	Males			Females		
	Farmers *n = 196*	Non-farmers *n = 208*		Farmers *n = 132*	Non-farmers *n = 139*	
	Median *(range)*		*p*	Median *(range)*		*p*
RBC ^1^ (10^6^·μL^−1^)	4.61	4.57	*0.588*	4.55	4.58	*0.275*
	*(3.65–6.75)*	*(3.74–6.75)*		*(3.74–6.52)*	*(3.65–6.14)*	
Haematocrit (%)	42.90	44.70	***<0.001***	38.95	41.00	***<0.001***
	*(29.20–59.90)*	*(33.90–54.60)*		*(23.40–52.20)*	*(30.60–49.20)*	
Haemoglobin (g·dL^−1^)	14.20	14.90	***<0.001***	12.60	13.60	***<0.001***
	*(10.40–19.60)*	*(11.30–18.20)*		*(7.30–17.90)*	*(10.20–16.40)*	
MCV ^2^ (fL)	89.20	89.40	*0.993*	90.30	89.20	*0.237*
	*(58.00–106.00)*	*(58.00–106.00)*		*(8.10–102.00)*	*(61.00–102.00)*	
MCH ^3^ (pg)	29.20	29.40	*0.471*	29.60	28.85	***0.014***
	*(18.40–34.80)*	*(18.90–35.10)*		*(18.90–32.50)*	*(18.20–34.30)*	
WBC ^4^ (10^3^·μL^−1^)	7.45	7.50	*0.849*	7.50	6.65	***0.010***
	*(3.50–17.00)*	*(3.70–17.00)*		*(3.10–13.50)*	*(3.10–13.50)*	
Neutrophils (10^3^·μL^−1^)	56.30	57.70	*0.253*	57.85	57.05	*0.174*
	*(28.10–78.90)*	*(5.90–84.70)*		*(3.00–79.50)*	*(3.00–77.10)*	
Lymphocytes (10^3^·μL^−1^)	30.15	32.00	*0.101*	32.95	31.00	*0.090*
	*(13.70–63.50)*	*(8.30–54.40)*		*(17.40–50.50)*	*(13.70–63.50)*	
Monocytes (10^3^·μL^−1^)	5.70	6.10	*0.192*	7.30	6.70	*0.181*
	*(1.80–14.40)*	*(1.80–13.80)*		*(3.10–12.60)*	*(2.50–14.40)*	
Platelets (10^3^·μL^−1^)	210.00	213.00	*0.426*	223.00	221.00	*0.591*
	*(10.00–365.00)*	*(63.00–377.00)*		*(65.00–377.00)*	*(121.00–376.00)*	

^1^ Red Blood Cells; ^2^ Mean corpuscular volume; ^3^ Mean corpuscular haemoglobin; ^4^ White blood cells.

**Table 4:** Biochemical blood examinations.

	Farmers *n = 328*	Non-farmers *n = 347*	
	Median *(range)*		*p*
Glucose (mg·dL^−1^)	97	95	*0.155*
	*(59–250)*	*(59–328)*	
Urea (mg·dL^−1^)	41	39	*0.369*
	*(13–98)*	*(13–98)*	
Creatinine (mg·dL^−1^)	0.8	0.8	*0.614*
	*(0.5–3.8)*	*(0.5–5.1)*	
Sodium (mEq·L^−1^)	139	139	*0.738*
	*(123–144)*	*(123–439)*	
Potassium (mEq·L^−1^)	4.3	4.3	***0.024***
	*(3.6–5.2)*	*(3.6–5)*	
Calcium (mEq·L^−1^)	9.3	8.9	***<0.001***
	*(8.4–10.2)*	*(8.4–10.2)*	
SGOT ^1^ (U·L^−1^)	40	35.0	***<0.001***
	*(9–95)*	*(9–55)*	
SGPT ^2^ (U·L^−1^)	33	33	*0.214*
	*(10–387)*	*(10–72)*	
γ-GT ^3^ (U·L^−1^)	34	35	*0.679*
	*(7–206)*	*(7–190)*	
LDH ^4^ (U·L^−1^)	186	169	***<0.001***
	*(112–428)*	*(112–257)*	
Amylase (U·L^−1^)	69	65	*0.250*
	*(24–133)*	*(24–125)*	
Alkaline phosphatase (U·L^−1^)	67	65	*0.140*
	*(26–137)*	*(26–116)*	
Total bilirubin (mg·dL^−1^)	0.8	0.7	***0.001***
	*(0.1–1.7)*	*(0.1–1.1)*	
Cholesterole (mg·dL^−1^)	169	166	*0.894*
	*(113–496)*	*(113–496)*	
Triglycerides (mg·dL^−1^)	116.5	110	***0.049***
	*(67–286)*	*(67–228)*	
Uric acid (mg·dL^−1^)	4.4	4.6	*0.320*
	*(2.6–8.1)*	*(2.6–8.1)*	
Total proteins (g·dL^−1^)	6.9	6.6	***<0.001***
	*(5.9–8.8)*	*(5.9–8.8)*	
CRP ^5^ (mg·dL^−1^)	0.6	0.4	***<0.001***
	*(0.1–1)*	*(0.1–0.8)*	
Pseudocholinesterase (U·mL^−1^)	7.7	7.7	***0.018***
	*(4.1–12.4)*	*(4.1–13.9)*	

^1^ Serum glutamic oxaloacetic transaminase; ^2^ Serum glutamic pyruvic transaminase; ^3^ Gamma-glutamyl transpeptidase; ^4^ Lactate dehydrogenase; ^5^ C-reactive protein.

### 3.5. Blood Pressure and Clinical Tests

**Figure 1:**
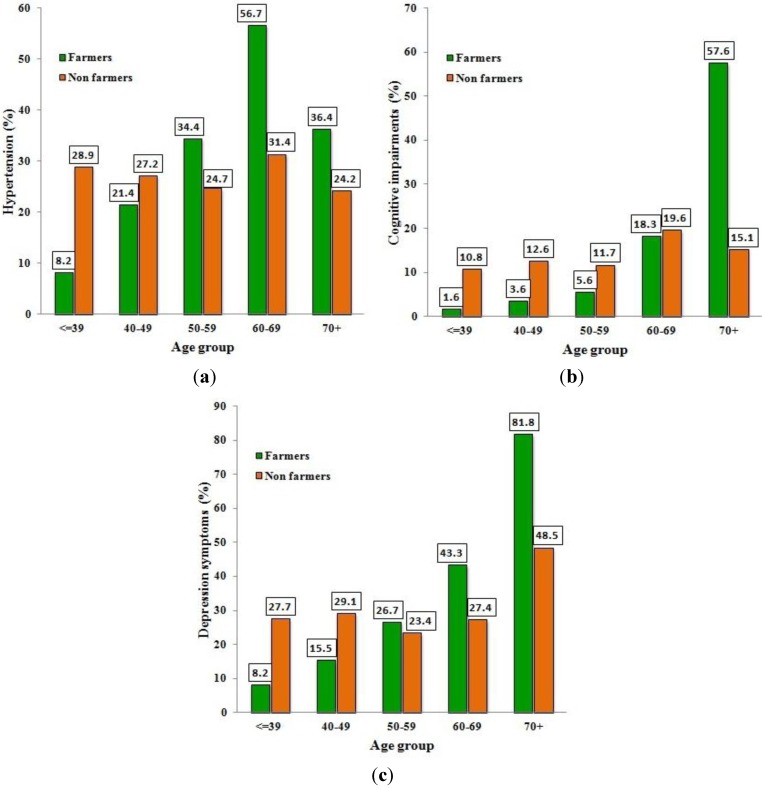
(**a**–**c**) Prevalence (%) of hypertension, cognitive impairments and depression symptoms in farmers and non-farmers per age group.

Since the prevalence of hypertension was increasing and the participants’ performances in neuropsychological tests were getting worse while aging, the corresponding classifications of subjects are depicted per age group in Figure 1(a–c).

As it is shown in Figure 1, the prevalence of hypertension, cognitive impairment and “any grade of depression” were lower for farmers than for non-farmers in the younger age groups while this observation was reversed in the older ages. 

The retained factors from multivariate logistic regression models of logit function for MMSE, MADRS and Hypertension are presented in Table 5.

**Table 5:** Multivariate logistic regression analysis.

		MADRS					MMSE					HYPERTENSION				
		N ^1^	Dep. ^2^				N ^1^	Cog. ^3^				No	Yes			
Factor	Coding	*n*	*n*	*β*	95% C.I. ^4^	*p*	*n*	*n*	*β*	95% C.I.	*p*	*n*	*n*	*β*	95% C.I.	*p*
**Occupation (Occ)**																
Non-farmers	Occ(0) *	246	101				301	46				252	95			
Farmers	Occ (1)	233	95	−0.755	−2.209–0.699	*0.309*	289	39	−1.857	−3.971–0.258	*0.085*	228	100	−1.591	−2.633–−0.549	*0.003*
**Gender (Gen)**																
Male	Gen (0) *	311	102				376	37				281	132			
Female	Gen (1)	168	94	−0.217	−0.781–0.347	*0.450*	214	48	0.539	−0.002–1.08	*0.051*	199	63	−0.822	−1.227–−0.417	*0.000*
**Age**						*0.199*					*0.912*					*0.975*
<40	Age (0) *	116	28				134	10				115	29			
40–49	Age (1)	144	43	−0.226	−0.910–0.457	*0.516*	171	16	0.114	−0.838–1.065	*0.815*	141	46	0.009	−0.652–0.669	*0.979*
50–59	Age (2)	125	42	−0.581	−1.342–0.180	*0.135*	153	14	−0.082	−1.122–0.958	*0.877*	117	50	−0.046	−0.770–0.678	*0.900*
60–69	Age (3)	71	40	−0.308	−1.133–0.518	*0.465*	90	21	0.397	−0.641–1.436	*0.453*	61	50	0.156	−0.627–0.939	*0.696*
70+	Age (4)	23	43	0.482	−0.411–1.374	*0.290*	42	24	0.049	−1.195–1.293	*0.938*	46	20	−0.185	−1.135–0.764	*0.702*
Gen * Occ																
Gen (1) by Occ (1)		168	94	1.103	0.326–1.880	*0.005*										
Age * Occ						*0.002*					*0.001*					*0.001*
Age (1) by Occ (1)		71	13	0.682	−0.661–2.026	*0.320*	81	3	0.338	−2.154–2.831	*0.790*	66	18	1.294	0.044–2.544	*0.043*
Age (2) by Occ (1)		66	24	1.500	0.139–2.862	*0.031*	85	5	0.791	−1.625–3.207	*0.521*	59	31	1.992	0.740–3.243	*0.002*
Age (3) by Occ (1)		34	26	2.156	0.732–3.580	*0.003*	49	11	1.920	−0.424–4.264	*0.108*	26	34	2.754	1.431–4.078	*0.000*
Age (4) by Occ (1)		6	27	2.846	1.189–4.503	*0.001*	14	19	3.631	1.166–6.096	*0.004*	21	12	2.280	0.769–3.791	*0.003*
**Number of children (Child)**																
0	Child (0) *	73	12			*0.010*	81	4			*0.014*					
1	Child (1)	52	11	0.166	−0.979–1.311	*0.776*	57	6	0.356	−1.009–1.720	*0.609*					
2	Child (2)	137	54	1.449	0.482–2.415	*0.003*	176	15	0.225	−0.993–1.443	*0.717*					
3	Child (3)	132	63	1.109	0.121–2.097	*0.028*	173	22	0.285	−0.923–1.493	*0.644*					
≥4	Child (4)	85	56	0.854	−0.184–1.892	*0.107*	103	38	1.218	0.038–2.399	*0.043*					
Ch.N. * Occ						*0.011*										
Child (1) by Occ (1)		17	3	−0.426	−2.448–1.597	*0.680*										
Child (2) by Occ (1)		76	15	−2.145	−3.712–−0.578	*0.007*										
Child (3) by Occ (1)		69	34	−1.417	−2.979–0.144	*0.075*										
Child (4) by Occ (1)		40	38	−0.562	−2.175–1.050	*0.494*										
**Income (Inc)**																*0.043*
<9,000 €	Inc (0) *						233	54			*0.010*	195	92			
9,000–15,000 €	Inc (1)						203	22	−0.710	−1.296–−0.125	*0.017*	168	57	−0.519	−0.941–−0.097	*0.016*
>15,000 €	Inc (2)						154	9	−1.001	−1.793–−0.208	*0.013*	117	46	−0.393	−0.865–0.079	*0.103*
**Smoking (Smok)**						*0.092*										
Smok (0) *	Νο	257	129													
Smok (1)	Moderate	105	20	−0.575	−1.157–0.006	*0.052*										
Smok (2)	Heavy	117	47	0.091	−0.428–0.611	*0.730*										
**Coffee (Coff)**																*0.122*
No Coffee	Coff (0) *											28	5			
1 cup /day	Coff (1)											44	29	1.226	0.110–2.342	*0.031*
2 cups/day	Coff (2)											311	125	0.802	−0.207–1.812	*0.119*
≥3 cups/day	Coff (3)											97	36	0.625	−0.435–1.684	*0.248*

^1^ N: normal. ^2^ Dep: any grade of depression. ^3^ Cog: cognitive impairments. ^4^ C.I.: confidence interval. * base category.

The results of Table 5 confirmed what is depicted in Figure 1. Specifically, MADRS score was influenced by occupation, gender, number of children and smoking. Odds Ratio (OR) for depression symptoms can be estimated for farmers *versus* (*vs*.) non-farmers after controlling for other characteristics. For instance, the OR for male farmers in the first age category (≤39 years) with no children *vs*. non-farmers with all the other characteristics being the same is the exponential of the coefficient of occupation *i.e*., OR = e^−0.755^ = 0.47. The corresponding OR for farmers in the age category 50–59 years old is the exponential of the sum of the occupation coefficient (−0.755) and the interaction coefficient of occupation x age category (1.500). The resulting OR is e^(−0.755+1.500)^ = 2.11, reflecting the worse scores of farmers in older ages. Furthermore, the coefficient of the interaction term occupation x gender indicates that the OR was increased for female farmers. The OR was increased with the number of children, but this factor acted protectively for farmers as it is indicated by the coefficients of the interaction term number of children x occupation. Additionally, moderate smoking (one pack per day) reduced the OR. 

As for MMSE, similar results were found, except that instead of smoking the income was retained into the model. The higher the income, the lower was the OR for cognitive impairments.

Hypertension was influenced by occupation, gender, income and coffee consumption. The ORs for farmers *vs*. non-farmers were lower than one in younger ages and got higher than one in the older ones. The ORs were lower in females compared to males. Similarly, the OR was reduced by income. Finally, consumption of two or more cups of coffee daily increased the OR. 

## 4. Discussion

The present study gives evidence of several clinical and neurobehavioral alterations among farmers who have been involved in cultivation for a long time-period, indicating their susceptibility to certain impairments of their health status in comparison with non-farmers living in the same area.

Based on the participants’ statements regarding their health problems, farmers mention more health issues than non-farmers at a statistically significant level (p = 0.044). Hypertension and other cardiovascular disorders occur more frequently among farmers in comparison to non-farmers. The results of blood pressure records confirm partially the personal statements given that the percentage of hypertension in younger ages is lower in farmers than in non-farmers of the same age, but this situation is reversed in older farmers (Figure 1(a)). Despite the intense physical activity of farmers, that is expected to act protectively, the results show a higher prevalence of hypertension in the older farming population. Elevated hypertension in farmers was also reported in a cross-sectional Australian study comparing farmers with the national averages [9]. In another study 21.5% of the studied Mexican migrant farmworkers in Arizona were found to suffer from hypertension while the corresponding percentage for Mexicans working in their own country was only 3% [22]. However, in other studies, lower morbidity rates and relative risks for cardiovascular diseases were found in farming populations [3,5]. 

In the present study, there is a higher frequency of orthopaedic problems in farmers, in accordance with previous studies that report injuries as the primary occupational health concern in agriculture [11,23]. A number of factors related to the nature of the agricultural work have been identified as possible risk factors for musculoskeletical and orthopaedic problems such as extensive workloads, heavy lifting, and working in stressful postures [10,22]. 

Farmers state that they experience more frequently ear, nose and throat disorders. Cough, pharyngitis, nasal catarrh, sinusitis, pharyngeal irritation and nasal irritation (dryness, sneezing and secretions) have been reported previously in a farming population in United Arab Emirates and were attributed to exposure to pesticides [24]. Moreover, self-reported hearing loss was found to be related to farm noise exposure [25].

The number of reported miscarriages among women farmers is slightly higher (on the borders of significance) in comparison with non-farmers. Previous studies with similar results have attributed that to pesticide exposure [26,27,28]. 

As far as the haematological blood examinations are concerned, a slight, still statistically significant, decrease in haematocrit and haemoglobin levels between both males and females farmers is observed, indicating possible suppression of heme biosynthesis. This finding is consistent with previous studies [29,30,31,32], and inconsistent with others [33]. Our results indicate an increase in WBC count among only female farmers, while no such increase is observed among male farmers. The findings from similar studies vary, as some have reported increased WBC activity [30,34], possibly due to stimulation of the immune system, while others have found no difference [33] or a slight decrease in WBC count [31]. 

Biochemical blood examinations revealed an increase in certain serum enzymes (SGOT, LDH and CRP) as well as in some serum components (total bilirubin, triglycerides, total proteins, calcium and potassium) accompanied by a simultaneous slight decrease in pseudocholinesterase levels in farmers compared to non-farmers. The raise of serum biochemical markers, which suggest liver dysfunction, or at least impairment of normal liver function, in those occupationally engaged to farming, has been previously reported [29,30,35], either by simultaneous alterations of all biomarkers [36], or via slight increases of certain components [34]. Total proteins levels were slightly higher among farmers in the present study, while other studies found no difference [35] or even lower total proteins levels in farmers [29,31] as a reflection of liver dysfunction. The increase in C-reactive protein (CRP) levels is an indicator of non-specific inflammation and oxidative stress in farmers’ population. Moreover the CRP elevation in farmers may contribute to an increased risk of hypertension, myocardial infarction and stroke as well [37,38,39]. As it can be seen from the results, plasma cholinesterase’s activity seems to be depressed among farmers, in accordance with previous studies showing significant decrease of serum cholinesterase [29,30,40] or erythrocyte acetylcholinesterase [34,41,42] activity among farmers or pesticide-producing workers [35].

As for the addressed neuropsychological tests, it is obvious that farmers in younger ages “start” with better scores than non-farmers. While farmers do achieve higher scores in MMSE when younger, they start to perform poorer than non-farmers during their middle-age and the differences become more obvious while aging. Respectively, young farmers gain lower scores and better performance in the MADRS test compared to young non-farmers, while this fact is reversed in aged subjects. 

The multivariate analysis (Table 5) confirms that the three health outcomes (hypertension, MMSE, MADRS scores) are better for farmers in younger ages and worse for those in older ages in comparison to non-farmers. This fact is stronger among females for the two neurobehavioral tests. It is worth to mention that the number of children is related with better MADRS performance for farmers in comparison with non-farmers. The above observations are retained after controlling for confounding factors, *i.e.*, education, income, number of children and coffee consumption. Taking into account the analyses results, the main outcome is that farming is related to hypertension, emotional burden and cognitive impairments. 

Most of the literature dealing with the health status of farmers worldwide presents contradictory findings. There are studies that measure better health in farming populations [1,2,3,4,6,11], while other studies argue for a worse health status in agricultural workers [9,10,22]. An issue that is under investigation is whether the better health of farmers reflects just the fact that rural populations have better health than urban ones, irrespective of occupation (urban-rural factor) or that is due to exposure to farming [1]. However, there is evidence that after controlling for the urban-rural factor, significant differences between the health status of rural farmers and non-farmers remain which may be attributed to other factors, such as exposure to farming, farmers' lifestyles, or other health-promoting factors [3,23]. 

The overall conclusion that can be drawn from the literature review is that even if farmers are “healthier” than the general population [2,3,11], they also demonstrate a distinct pattern of specific disease prevalence, namely unintentional fatal injuries, certain types of cancer, chronic respiratory diseases, liver diseases and cognitive and emotional disorders as reflected by their higher suicides rates [10,11]. The present study is consistent with the above mentioned findings and highlights specific physical and mental impairments observed on farmers while aging. Undoubtedly farming induces stressful conditions. Feelings like isolation and uneasiness aggravate common depressive symptoms that other people may have been able to cope with. All over the world the consequences of an industrial approach to enhance the crops along with the farming crisis have deprived many small farmers of their self-sufficiency and urge them into despair, thus increasing the reported mood disorders. It has been referred that social support and sense of belonging are protective factors for psychological disorders in farmers [43].

The main limitation of the study arises from its nature. The measures of association that can be estimated by a cross-sectional study are Odds Ratios and no causal inferences can be drawn. Nevertheless, the findings of the cross-sectional studies are a good starting point for further investigation. Another issue of concern is the “healthy worker effect”. However, that seems not to be the case for the present study since young farmers have better health indices than non-farmers, at least for the three main outcomes. Additionally, male farmers are wealthier than non-farmers. Another possible limitation is the potential misclassification of self-reported morbidities, though these were concurrent with the literature. Despite these limitations, this study still managed to discern the discrepancies between farmers and non-farmers in various age groups. 

What remains to be clarified is which are the factors that contribute to this unique health pattern that is presented into the farming population. Farming is a valuable resource worth preserving. Policies for supporting the agriculture workers are necessary globally but especially in countries such as Greece, in which economy is closely related to agriculture.
